# Notes on Syphilis

**Published:** 1874-02-01

**Authors:** 


### Original Translations.

### NOTES ON SYPHILIS.

*Translated for* THE EXAMINER, *from Le Progres Medical of Sept. 27th, and Oct. 11th*, 1873.

SYPHILITIC ULCERATIONS OF THE CERVIX UTERI.—Dr. A. Le Blond, in discussing these lesions, describes:

1st, *The Chancre.* — The soft variety is most frequently encountered in this location. The indurated sore, though actually observed, is regarded as of rare occurrence. M. Alphonse Guerin, though not denying the possibility of induration, considers that it can rarely be discovered by digital examination. M. Armand Despres, (on "Ulceration and Ulcers of the Cervix Uteri," Paris, 1869, p. 46) believes that induration never occurs in this situation; but this statement is contradicted by Ricord's observation of the fact, in a case where the cervix projected beyond the vulva. Soft chancres of the cervix are not only the more frequent, but are often succeeded by constitutional infection. They occur in the form of adherent ulcers, having a grayish base, irregular borders, and clean-cut edges, surrounded by a somewhat inflamed areola. They are generally multiple, almost always coalesce after a certain interval, and may become phagedenic. Bernutz (on "Syphilitic Affections of the Uterus," *Union Medicale*, 1855, p. 275) describes a variety of sore which he designates, "the diphtheritic chancre," characterized by a yellowish-gray secretion adherent to the base of the ulcer, and limited by projecting, red and clean-cut edges.

The same author describes a form of ulcerative chancre which is of rare occurrence. This species of sore invades the uterine cavity and excavates its tissues, very much as those ulcers of the male sex, which encroach upon the canal of the urethra.

The chancre is habitually seated, not at the summit of the neck, as is the case with simple ulceration, but at a variable point, and principally, as M. Marjolin remarks, at the point of union of the vagina and cervix. In certain cases, according to Bernutz and Courty, the chancre is found in the canal of the cervix, and dilatation is requisite for its discovery.

The characters herein assigned to the different varieties of chancres are liable to be lost after a certain interval; and then they may present the appearance of simple inflammatory ulceration, mucous condylomata, or vegetations, resting upon a slightly indurated base, which might lead to a suspicion of cancerous degeneration. In these cases, the march of the disease is the sole criterion for accurate diagnosis.

In certain cases, the chancrous inoculation occurs at a point where there has been previous inflammatory ulceration; the chancre then loses its distinguishing features; but the surface of the ulcer generally becomes somewhat grayish, soft and fungous.

2d, *Mucous Patches.* — The second species of syphilitic lesion, occurring at or near the cervix uteri, is the "mucous patch," which is distinguished by an elevation of the ulcerated surface above the level of the mucous membrane upon which it is seated, the latter being of a pearly-white color. One of the chief features of these lesions is their contagiousness. After persistence for a variable period, under the form here described, they ordinarily change their features, and are not to be distinguished from simple ulcers.— *For the Author's appendix to his translation of Churchill's Diseases of Women.* 2d edition.

Syphilis and Rachitis of the New Born.—M. Parrot considers that there are three essential characters of fully developed rachitis: 1st, decalcification; 2d, formation of spongy tissue; 3d, medullization. Decalcification affects all the bones, which become friable. The formation of spongy tissue presents the appearance of red granulations. Medullization occurs at the level of the spongy tissue, and in the case presented, affected the cartilaginous layer. If the cranium be examined, it will be found that the spongy tissue is in excess externally, in proportion as it is deficient internally. This fact gives us a point of departure from which we can proceed to distinguish the peculiarities of syphilitic bones in newly-born infants, and in those under one year of age.

Infants affected with this disease, have specific lesions of peculiar and different characters:

1st *Period.*—Very marked in children from one day to six weeks old. There is: (*a*) Exuberance of calcification at the extremities of the long bones; a chondro-calcareous zone, and peri-diaphyseal osteophyma, from about two-fifths to four-fifths of a line in thickness; (*b*) Gelatiniform degeneration of pre-existent tissues, involving both the cartilaginous and spongy portions. The bones are denser and more difficult of section than in normal conditions. The detachment of the epiphyses sometimes established, is not common to rachitis and syphilis.

2d *Period.*—Children from six weeks to several months old. (*a*) Occurrence of phenomena described above: osteophyma and gelatiniform degenerations; (*b*) Medullization and decalcification attack the primitive bone and the osteophyte. These modifications are well marked. There is, hence, a certain resemblance to rachitis, where these lesions are universally more pronounced. The *specificity* is soon not to be distinguished; the toxæmia becomes less evident; an ordinary cachexia gradually results. For the purpose of diagnosis, the following summary of differential features is appended:

| RACHITIS. | SYPHILIS. |
|---|---|
| Spongy tissue. | No spongy tissue. |
| Peripheric, spongy layers. | Layers of osteophytic bone. |
| Increase of diameter by formation of spongy tissue much less considerable. | Increase of diameter by osseous neoplasmic layers marked: (Inferior extremity of humerus; middle portion of diaphysis of tibia.) |
| Medullization and decalcification, considerable. | Medullization and decalcification scarcely noticeable. |
| No gelatiniform degeneration. | Gelatiniform degeneration sufficient for detachment of epiphyses. |

3d *Period.*—It is, at this time, difficult to establish a distinction. An intimate knowledge of the first two periods is requisite. The lesions connected with the first tend to disappear; those which are common to syphilis and rachitis become exaggerated. Spongy tissue is to be found more frequently at the periphery, and less often at the extremities. Subjoined is a table of differences:

| RACHITIS. | SYPHILIS. |
|---|---|
| No osteophytic layers. | Osteophytic layers, with interposed medullary lacunæ. |
| Increase of diameter by formation of spongy tissues only. | Increase of diameter by osteophytic layers, and by spongy tissue. |
| Bones more flexible. | |

Evidently, it is the primitive characteristics which persist, and which must be the foundation for diagnosis. These are wanting in those bones where osteophytic layers are not formed. On the other hand, these layers may be completely destroyed by medullization. In such cases, certain bones, at a point of election (inferior extremity of the humerus) would present a considerable increase in thickness. We have never met with such cases, but must admit, theoretically, that they might occur. The rachitis would doubtless soon mask the syphilis. The toxæmia gradually subsiding, would, by degrees, lose its external traces; it would be overpowered in the rachitic cachexia. This is a species of morbid transformation —of pathological hybridity. The syphilis has, in some way, summoned to its side the rachitis, which commenced by combining with it, and concluded by gradually absorbing it for its own use and profit.

ABSENCE OF LESIONS OF VISCERAL SYPHILIS IN CASE OF DEATH FROM PYÆMIA.—*Discussion.*—The patient, G., fifty-one years old, had had, in youth, symptoms of scrofulous disease of bones; typhoid fever and syphilis in 1868 (papular syphilides, mucous patches, iritis, traces of former ulceration of the neck).

A vegetating villous tumor, as large as an apple, whose pedicle was attached to the posterior lip of the cervix, was removed June 21, 1873, by M. Despres, with the ecraseur. Death occurred in consequence of septicæmia, June 28th.

Autopsy made thirty-six hours after death, revealed evidences of decomposition, excessive tumefaction of abdomen, and discoloration of integument, lungs, spleen, kidneys and liver, which were full of blood and friable. In the lungs, numerous metastatic abscesses were found, but none in the liver.

The noticeable fact in the case was, that the woman, though syphilitic five years before, presented no hepatic cicatrices. The capsule of Glisson was intact, and the surface of the liver polished and smooth.

In the vesico-uterine cul-de-sac, about 150 grammes of a sero-purulent liquid were discovered. The posterior wall of the womb was somewhat thickened, and, on section, the orifices of the uterine sinuses, widely opened, gave issue to a thick and creamy pus. The wound produced by the removal of the epithelioma, exhibited pits, from which, also, some drops of pus could be expressed.

The left cervico-vaginal pouch was thickened, indurated, and united with the neighboring cellular tissue; the uterine vessels, which were prolonged into this altered mass, were injected with pus. On the right side, the conditions were normal.

*M. Despres* remarked: The facts of this case are negative as regards the lesions of visceral syphilis; but I have recently had occasion to examine the body of a non-syphilitic patient, which presented numerous cicatrices of the liver, consecutive to the resorption of several hydatid cysts of that organ.

*M. Charcot.*—Such facts prove nothing as against the doctrines of visceral syphilis. The question rests precisely where M. Despres found it. It can always be objected, and with reason, that syphilis is fortunately not fatally injurious to the viscera; and, on the other hand, this should not invalidate the claim of other cases, in which a distinctive influence has been brought to bear upon the hepatic gland. The influence of syphilis in the production of exostoses is undeniable; and yet such lesions are not invariably discovered after death. This mode of reasoning is, therefore, extremely open to criticism.

*M. Despres.*—I insist upon the existence of a unity of facts which, when added to other unities, has the effect of a demonstration. Once, the discovery of hepatic cicatrices was sufficient to establish a diagnosis of *ante-mortem* syphilis. Cases have even been cited where cicatrices existed two years after the initial chancre. Medical belief seems about to return to these evident exaggerations. In the same way, every hypertrophy of the spleen, found under analogous circumstances, is claimed to be syphilitic. Now, the present case demonstrates the absolute falsity of these assertions, since the spleen is not enlarged.

*M. Landouzy.*—This last fact has not all the importance assigned to it by M. Despres. Because splenic hypertrophy is of diagnostic value in syphilis, it does not follow that a normal volume of that organ is proof positive against the existence of constitutional syphilis.

In Frerichs, Leudet, and Lancereaux there are reported twenty-eight cases of splenic hypertrophy in adults, manifestly syphilitic. In twenty-seven of these, the hepatic lesions were constant, profound, and classified as follows: Retraction, deformity, cicatrices, nodosities and cirrhosis. Cases of syphilitic megalo-splenia, coincide, then, with internal lesions of the liver; the second seem to command the first, as, in the drunkard's liver, and the cardiac liver, the splenic hypertrophy succeeds to the disturbance of the hepatic circulation.

If splenic enlargement has a certain value in syphilis, it certainly does not seem proper to argue anything from its absence; since we may have cutaneous, osseous, or visceral lesions of syphilis, without splenic complication, this organ appearing, generally, to become implicated only when profound and extended lesions of the liver seriously derange the portal circulation.

*M. Charcot.*—The question is to be decided, not by discussion, but by facts. Does M. Despres admit the anatomical specificity of the syphiloma, or of the gummy tumor of the liver? I have often discovered, in the parenchyma of the liver, lesions corresponding to the descriptions of Virchow and Wagner. The question of cicatrices is different. These may be the result of retrogressive hydatid cysts, traumatic injury, and even, according to Virchow, of atrophic cancer. The diagnosis must necessarily be difficult; but it does not result that we must reject the possibility of gummy tumors of the liver, because every hepatic cicatrix is not necessarily of syphilitic origin.

*M. Despres.*—In an anatomical point of view, the so-called syphilomata are not to be distinguished from the lesions of lymphadenia and tuberculosis; and this is avowed by Virchow, who cites appropriate cases. In the majority of cases reported by M. Lancereaux, we discover the simultaneity of pulmonary tubercles. The question, then, arises: Is not the so-called syphiloma of the liver a tuberculous manifestation, or, rather, an evidence of modified syphilis in a tuberculous subject? I deny, absolutely, the existence of gummy tumors of the liver as specific products.

The president of the Anatomical Society (M. Charcot), closed the discussion by inviting its members to return with facts either substantiating or disproving the position taken by M. Despres. J. N. H.

